# Molecular Diagnostics, Targeted Therapy, and the Indication for Allogeneic Stem Cell Transplantation in Acute Lymphoblastic Leukemia

**DOI:** 10.1155/2011/154745

**Published:** 2011-11-10

**Authors:** Anthony Oyekunle, Torsten Haferlach, Nicolaus Kröger, Evgeny Klyuchnikov, Axel Rolf Zander, Susanne Schnittger, Ulrike Bacher

**Affiliations:** ^1^Department for Stem Cell Transplantation, University Cancer Center Hamburg (UCCH), 20246 Hamburg, Germany; ^2^Department of Hematology and Immunology, Obafemi Awolowo University (OAU), Ile-Ife, Nigeria; ^3^MLL Munich Leukemia Laboratory, Munich, Germany

## Abstract

In recent years, the panel of known molecular mutations in acute lymphoblastic leukemia (ALL) has been continuously increased. In Philadelphia-positive ALL, deletions of the *IKZF1* gene were identified as prognostically adverse factors. These improved insights in the molecular background and the clinical heterogeneity of distinct cytogenetic subgroups may allow most differentiated therapeutic decisions, for example, with respect to the indication to allogeneic HSCT within genetically defined ALL subtypes. Quantitative real-time PCR allows highly sensitive monitoring of the minimal residual disease (MRD) load, either based on reciprocal gene fusions or immune gene rearrangements. Molecular diagnostics provided the basis for targeted therapy concepts, for example, combining the tyrosine kinase inhibitor imatinib with chemotherapy in patients with Philadelphia-positive ALL. Screening for *BCR-ABL1* mutations in Philadelphia-positive ALL allows to identify patients who may benefit from second-generation tyrosine kinase inhibitors or from novel compounds targeting the T315I mutation. Considering the central role of the molecular techniques for the management of patients with ALL, efforts should be made to facilitate and harmonize immunophenotyping, cytogenetics, and molecular mutation screening. Furthermore, the potential of high-throughput sequencing should be evaluated for diagnosis and follow-up of patients with B-lineage ALL.

## 1. Introduction

Acute lymphoblastic leukemia (ALL) is a heterogeneous disorder, which consists of various clinical, morphological, and immunological phenotypes, underpinned by extreme genetic diversity [2–4]. Adaptation of treatment intensity to the probability of relapse in the individual patient requires a thorough understanding of the risks represented by the various stratified leukemia subtypes. This has been achieved, to a large extent, using a broad spectrum of diagnostic techniques including cytomorphology, immunophenotyping, cytogenetics, fluorescence in situ hybridization (FISH), and molecular techniques. The panel of known prognostically important molecular alterations is constantly increasing, as demonstrated by the recent detection of alterations of *TGF-beta* and *PI3K-AKT* pathway genes and prognostically adverse deletions at 6q15-16 in T-ALL [5]. In Philadelphia-positive (B-lineage) ALL, deletions of the *IKZF1* gene confer a more adverse prognosis [6, 7]. Genetic alterations are now detectable in most ALL patients, when cytogenetic and molecular techniques are combined. These genetic alterations are linked to distinct clinical profiles and show specific interaction with other mutation types [8]. Following the success of the tyrosine kinase inhibitor (TKI) imatinib in chronic myeloid leukemia (CML), research focused on targeted therapy strategies for Ph-positive ALL and other ALL subtypes [9–13]. Imatinib has since become part of pre- and posttransplant treatment for patients with Ph-positive ALL [13, 14]. Rituximab was included in treatment of CD20-positive ALL [15–17]. This paper characterizes the most important molecular markers in patients with acute lymphoblastic leukemia, paying attention to their impact for treatment decisions, and discusses methods for their detection. 

## 2. B-Lineage Acute Lymphoblastic Leukemia (ALL)

**Figure 1:**
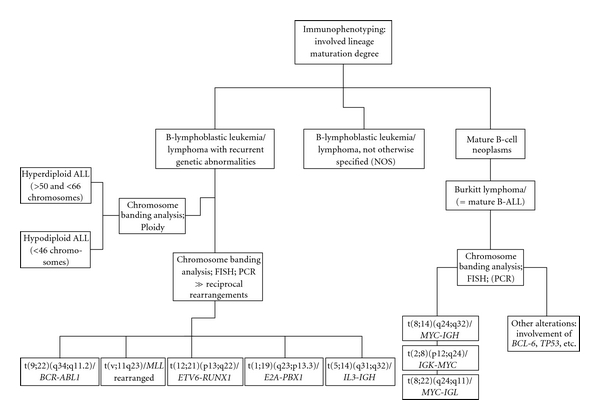
Classification of different B-lineage ALL/LBL entities according to WHO, 2008 [1].

According to the WHO classification published in 2008 [1], different reciprocal rearrangements form the category “B-lymphoblastic leukemia/lymphoma with recurrent genetic abnormalities” (Figure 1). Many of these genetic alterations provide useful markers to monitor the minimal residual disease (MRD) load [18]. 

### 2.1. Philadelphia-Positive ALL

In Ph-positive ALL, the t(9;22)(q34;q11.2)/*BCR-ABL1* can be detected with chromosome banding analysis in 95% of cases, but due to chromosome preparation, there is a latency of some days until results are available, and the *BCR-ABL1* rearrangements are cryptic in around 5% of all cases. Thus, interphase FISH or PCR for *BCR-ABL1* should be performed in every case of B-lineage ALL. Since imatinib has been added to intensified chemotherapy [19], prognosis of this previously highly adverse subgroup has been significantly improved. RT-PCR analysis allows a correct detection and classification of all cases according to the breakpoints (*m-BCR* in the majority of cases; *M-BCR* in ~30% of cases). Deletions of the *IKZF1* gene confer an adverse risk profile in Ph-positive ALL [6, 7]. The *IKZF1* gene has a coding function for a transcription regulator involved in T- and B-cell differentiation.

### 2.2. Burkitt Lymphoma/Mature B-ALL

Burkitt lymphoma/mature B-ALL is part of the category “mature lymphatic neoplasms” according to the revised WHO classification [1]. The most frequent is the t(8;14)(q24;q32)/*IGH-MYC* rearrangement [20]. Interphase FISH detects the diverse *MYC *rearrangements irrespective of the involved partner chromosomes, but can as well identify specific *MYC* rearrangements. PCR is less suitable for this purpose due to the heterogeneous breakpoints. The large and rapidly increasing tumor burden in Burkitt lymphoma can progress quickly to cause life-threatening complications and thus requires immediate therapeutic intervention. Therefore, interphase FISH analysis screening for *MYC *rearrangements should be performed without delay in all suspicious cases (highly elevated LDH, strongly basophilic and vacuolated cytoplasm of the lymphoblasts, or a large lymphoma load which developed over a short time span). As endemic EBV-related Burkitt lymphoma occurs most commonly in malaria-endemic and resource-poor areas, where facilities for FISH may be unavailable, characteristic morphologic appearances on cytology and histology still have an important role for diagnosis of this particular lymphoma subtype. 

### 2.3. Other Recurrent Mutations in B-Lineage ALL

The most frequent *MLL *rearrangement in ALL is the t(4;11)(q21;q23)/*MLL-AFF1*, but various other partner genes that can rearrange with *MLL*/11q23 have been identified [21]. In general, 11q23/*MLL* rearrangements confer adverse prognostic implications, just as in AML [1]. The search for the *MLL *rearrangements can be done with interphase FISH, while RT-PCR can be deployed to detect many specific rearrangements. The t(1;19)(q23;p13)/*E2A-PBX1* translocation characterizes 25% of pediatric precursor B-lineage ALL and confers a poor prognosis. 

In pediatric B-lineage ALL, the prognostically favorable t(12;21)(p13;q22)/*ETV6-RUNX1 *(*TEL-AML1*) fusion is the most frequent recurrent translocation and occurs in approximately 25% of precursor B-lineage ALL cases. The respective gene fusion cannot be detected with chromosome banding analysis, whereas interphase FISH or RT-PCR can reveal this reciprocal rearrangement without difficulties. Screening for the respective gene fusion is mandatory in children with B-lineage ALL as it confers a favorable prognostic impact [22]. 

Kuiper et al. performed an evaluation of risk parameters in pediatric patients with precursor B-lineage ALL. In a multivariate model, the presence of *IKZF1* deletions remained the strongest predictive factor for relapse-free and overall survival (*P* < 0.001), thereby surpassing previously identified prognostic factors, including the presence of *BCR-ABL1* gene fusions, DNA index, age, and white blood cell count [23].

## 3. Targeted Strategies in B-Lineage ALL

### 3.1. Tyrosine Kinase Inhibitors 

Following the success of imatinib in CML, TKIs were evaluated for *BCR-ABL1*-positive ALL. Concurrent or alternating combination of imatinib with intensive chemotherapy for remission induction and consolidation was able to achieve morphologic remission in 95–100% and molecular remission in ~50% of adults with Philadelphia-positive ALL [12–14, 24]. Outcomes were significantly improved as compared with historical controls who received similar chemotherapy regimens but no imatinib [25]. Presently, imatinib combined with chemotherapy is standard for Ph-positive ALL proceeding to a possible transplantation [26]. Since most adult patients would relapse after chemotherapeutic treatment alone, allogeneic HSCT is still being recommended for adult patients with Philadelphia-positive ALL in first CR [25]. Also in the posttransplant period, imatinib has been integrated for prophylactic reasons [11]. 

Other options for Ph-positive ALL include the use of second-generation TKIs, which have higher *BCR-ABL1* affinity and are effective in many patients with resistance to first-generation TKIs, for example, due to de novo variant *BCR-ABL1* isoforms or imatinib resistance-conferring mutations at the *BCR-ABL1* kinase domain. Ottmann et al. evaluated the success of dasatinib in 36 patients with Ph-positive ALL who were refractory or intolerant to imatinib. Major hematologic responses (MHRs) were achieved in 42% of patients with a median interval to MHR of 1.8 months. Among patients who achieved MHR, response duration ranged up to 8.7 months. Ten of the 15 patients (67%) who achieved an MHR remained free of progression at the 8-month follow-up. Complete cytogenetic responses were attained by 58% of patients. Only 6% of patients discontinued therapy as a result of study-drug toxicity [27]. 

Unfortunately, the multi-TKI-resistant T315I mutation develops more frequently and relatively faster in patients with Philadelphia-positive ALL than in patients with chronic phase of CML who receive TKI treatment [28, 29]. In a recently concluded phase I clinical trial, the multikinase and pan-*BCR-ABL1* inhibitor, ponatinib (AP24534) induced a complete cytogenetic and major molecular response rates of 89% and 78%, respectively, in CML patients with T315I, and most responses were maintained after 12 months of follow-up [30, 31]. However, it remains to be seen if these responses will be confirmed. Additionally, DCC-2036, a new TKI in a novel class of so-called “switch pocket inhibitors,” is undergoing trials for patients who carry T315I or who have failed TKI treatment. DCC-2036 targets a pocket that governs transition to the active state of ABL1, thus locks the kinase into its inactive state through a selective, non-ATP-competitive mechanism [30, 32]. GNF-2, another new agent, inhibits the T315I kinase by binding to the autoregulatory allosteric myristate cleft at the N-terminus of ABL1, also effectively freezing the kinase in its inactive state [30, 33]. These new compounds represent interesting new options for patients with *BCR-ABL1*-positive leukemias.

### 3.2. Monoclonal Antibodies with Anti-CD20 Activity

Rituximab is a chimeric monoclonal antibody directed at the (B cell) CD20 receptor. Its activity is associated with induction of antibody-dependent cytotoxicity, or direct apoptosis [34]. As the CD20 antigen is frequently expressed in B-lineage ALL, rituximab has been successfully combined with intensive chemotherapy regimens in B-lymphatic neoplasms of low- and of high-grade malignancy. Thomas et al. suggested the inclusion of rituximab into a modified hyper-CVAD regimen (cyclophosphamide, vincristine, doxorubicin, and dexamethasone) for adolescents and adults with de novo precursor B-lineage ALL. In patients with CD20 expression, rituximab improved outcomes compared with the historical experience using hyper-CVAD alone, with 3-year CR duration rates of 68% versus 28% in the historical cohort (*P* < 0.001) [16]. In mature B-ALL (Burkitt lymphoma), survival rates increased >80% with the combination of short intensive chemotherapy and rituximab [19]. Rituximab can also be used for intrathecal therapy for CD20-positive ALL patients with CNS disease failing to respond to intrathecal chemotherapy [17]. In the allogeneic transplant setting, Kebriaei et al. incorporated rituximab in the conditioning regimens for adolescents and adults with CD20-positive ALL [35]. 

### 3.3. Monoclonal Antibodies with Anti-CD19 Activity

Topp et al. just recently reported a phase II study in which the efficacy of the bispecific single-chain anti-CD19 antibody blinatumomab was studied [36]. The drug was administered to 21 B-lineage ALL patients with MRD persistence or relapse after-chemotherapy. Sixteen patients (76%) responded and became MRD negative. Estimated relapse-free survival at a median follow-up of 405 days was 78%, and the most frequent severe adverse effect was a reversible lymphocytopenia. The authors concluded that blinatumomab is efficacious and well tolerated in this subgroup of patients, after intensive chemotherapy. It was noted that T cells engaged by blinatumomab seemed capable of eradicating chemotherapy-resistant tumor cells [36].

### 3.4. Indication for Allogeneic HSCT in B-Lineage ALL

Conventional practice dictates that ALL patients in 2nd complete remission (CR2) or beyond invariably require allogeneic HSCT [37, 38]. Likewise, patients with high-risk disease are recommended for HSCT in CR1. However, on account of the good results recently reported for Ph-positive ALL with the tyrosine kinase inhibitors, there may be a need to reevaluate the “risk” status of the Philadelphia chromosome in ALL [10, 39–41]. The GRAAPH study group had examined imatinib-intensified chemotherapy and HSCT in 45 adult Ph-positive ALL patients and reported an overall CR rate of 96%. Among the 22 patients who had donors and received allogeneic HSCT in CR1, the estimated cumulative incidences of relapse, disease-free survival, and overall survival were 30%, 51%, and 65%, respectively. These end points compared very favorably with results obtained in the pre-imatinib era [12]. The JALSG prospectively treated 80 adult Ph-positive ALL patients with imatinib-fortified chemotherapy and reported a CR rate of 96%. Allogeneic HSCT was performed for 49 patients. Among the current trial patients, the probability for OS at 1 year was 73.3% for the recipients of allogeneic HSCT, and 84.8% for patients without HSCT [13]. Schultz et al. evaluated whether imatinib with an intensive chemotherapy regimen improved outcome in 92 children and adolescents with Ph-positive ALL and compared toxicities to 65 Ph-negative ALL patients given the same chemotherapy without imatinib. Three-year EFS was similar for patients in the cohort treated with chemotherapy plus imatinib (88%  ± 11%) or sibling donor BMT (57%  ± 22%). There were no significant toxicities associated with adding imatinib to intensive chemotherapy [10]. Thus the outcomes for patients with Ph-positive ALL treated with imatinib-containing chemotherapy were becoming more like those for patients with standard risk ALL.

For patients with critical ALL subtypes, it remains to be seen whether future MRD strategies might focus on subclone analysis and on tracking all minor and major clones during the early phases of chemotherapy [42]. This might result in a higher reliability to predict the relapse risk and might contribute to identify those patients who might benefit from an early allogeneic HSCT. 

## 4. T-Lineage Acute Lymphoblastic Leukemia (T-ALL) 

Clonal cytogenetic anomalies are detectable in 50–70% of all cases of T-cell ALL. Reciprocal translocations usually involve the T-cell receptor (TCR) genetic loci; *TCRA* and *TCRD* (14q11.2), *TCRB* (7q35), or *TCRG* (7q14-15). The partner genetic loci reported are usually transcription factors particularly *HOX11* (*TLX1*, 10q24), *HOX11L2* (*TLX3*, 5q35); others include the *MYC* (8q24.1) or *TAL1* (1p32) genes. Other fusion genes are, for example, *CALM-AF10* or *NUP214-ABL1*. For molecular MRD measurement, suitable fusion transcripts are available for only 10–20% of T-ALL patients. If appropriate targets are available, quantitative real-time PCR can achieve sensitivity of 10^−4^ to 10^−5^. In the alternative, clone-specific TCR rearrangements of the leukemic T cells could equally serve for MRD monitoring in remission with comparable sensitivity. However, amplification of clone-specific TCR rearrangements is highly laborious as patient-tailored assays are required. Furthermore, molecular clonal evolution can lead to false-negative results [43].

### 4.1. Therapeutic Strategies in T-Lineage ALL

Although current treatment protocols result in complete remission in 80–90% of adults with newly diagnosed T-cell acute lymphoblastic leukemia (T-ALL) or lymphoblastic lymphoma (T-LBL), approximately half of these patients relapse within the first two years [44]. The prodrug nelarabine is demethylated by adenosine deaminase to a deoxyguanosine derivative (ara-G). DeAngelo et al. administered nelarabine to 26 patients with T-ALL and 13 with T-LBL who were refractory to at least one multiagent regimen or had relapsed. Cycles were repeated every three weeks. The complete remission rate was 31%, and the 1-year overall survival was 28%. The overall tolerability was acceptable [45, 46]. Due to the clear antitumor activity in relapsed/refractory T-ALL/T-LBL, the compound has been approved by the FDA for patients who failed at least in two prior regimens [47]. 

In comparison to B-lineage ALL, it is more difficult to clarify the prognostic meaning of karyotypes in T-lineage ALL due to the lower incidence. Normal karyotypes and the t(10;14)/*HOX11-TCR* were shown to be associated with good outcomes in pediatric T-ALL [48]. 

### 4.2. Indication for Allogeneic HSCT in T-Lineage ALL

The use of conventional ALL chemotherapy for T-cell ALL has been associated with inferior outcomes compared to B-cell ALL, and thus most T-cell ALL were considered high risk. However, there have been suggestions of improved outcomes with more aggressive use of antimetabolite therapy in T-ALL subgroups [49], largely because these lymphoblasts accumulate methotrexate polyglutamates less avidly than blasts of other subtypes [50]. In the pediatric setting, Schrappe et al. had indeed shown clinically that high-dose methotrexate is associated with improved outcomes in T-cell ALL [51]. Similarly, Pui et al. used increased doses of methotrexate in the 76 pediatric patients diagnosed with T-ALL and also achieved improved outcomes, with estimated 10-year survival rate of 90% [52]. The indication for allogeneic stem cell transplantation in the first remission of T-lineage ALL is based on the individual risk profiles defined, for example, by the immunophenotype. Thymic (or cortical) T-ALL is considered to represent standard risk leukemia, whereas early and mature T-ALL confers high risk. Other than that, nonresponse to induction and consolidation regimens or increase of the MRD load during the course of disease can be indications to allogeneic transplantation.

## 5. Monitoring of the Minimal Residual Disease (MRD) Load

After patients achieve complete remission following either chemotherapy or HSCT, the MRD load should be serially assessed [53]. It is thus desirable to identify a sufficiently specific leukemia-specific marker before-therapy, such as the *BCR-ABL1* fusion. The preferred MRD technique depends on the desired level of sensitivity or the depth of remission. Cytogenetics has a sensitivity of 10^−2^ cells. Interphase fluorescence in situ hybridization (FISH) allows to evaluate 100–200 cells. Immunophenotyping using multi-parameter flow cytometry achieves sensitivity levels of 10^−3^ to 10^−5^ [54, 55]. Real-time PCR is particularly useful, as it can achieve a sensitivity of 10^−4^ to 10^−6^ [56]. Additionally, molecular techniques can be used to access MRD in ALL even in the absence of fusion genes, by assessing the levels of clone-specific rearrangements of the immunoglobulin or T-cell receptor [57] and have been introduced into treatment stratification already. In a study from the German Multicenter Study Group for Adult Acute Lymphoblastic Leukemia (GMALL), a total of 196 patients with standard risk ALL were investigated at repeated time points in the first year by quantitative PCR monitoring of clonal immunoglobulin or *TCR* rearrangements. Three risk groups could be defined. Patients with a rapid decline of the MRD load to <10^−4^ or below detection limit in the early treatment period (days 11 and 24) were classified as low risk and had a three-year relapse rate of 0%. Patients with an MRD of ≥10^−4^ until week 16 formed the high-risk group with a 3-year relapse rate of 94%. The remaining patients had an intermediate risk [58]. In another study from the GMALL, postconsolidation samples of 105 patients with standard risk ALL were investigated by real-time quantitative PCR for clonal immune gene rearrangements. All patients were beyond the first year of chemotherapy, in hematological remission, and were MRD negative before study entry. The relapse rate was 61% in patients converting to MRD positivity thereafter, whereas only 6% of continuously MRD-negative patients relapsed [59]. 

Expert panels have already suggested recommendations on the minimal technical requirements before implementation of MRD diagnostics into clinical trials and have standardized criteria for “complete MRD response,” “MRD persistence,” and “MRD reappearance.” These steps facilitate the comparison of MRD results between different treatment protocols [60]. The determination of B-cell specific donor chimerism may facilitate monitoring and therapeutic decisions in patients with B-lineage ALL in the posttransplant period [61]. 

## 6. Conclusion

In recent years, molecular diagnostics in the acute lymphoblastic leukemia have progressed rapidly. PCR-based analyses in combination with other approaches (cytogenetics, FISH, and immunophenotyping) have allowed us to define various distinct ALL subtypes, part of which already defines separate entities within the WHO classification of 2008, for example, the t(9;22)/*BCR-ABL1* or the t(12;21)(p13;q22)/*ETV6-RUNX1*. Deeper insights into the networks of molecular markers have facilitated the understanding of the heterogeneity of the clinical courses within distinct genetic subgroups and improved therapeutic decisions, for example, regarding the indication to allogeneic HSCT within T-lineage ALL [49]. Screening for deletions of the *IKZF1* gene might improve risk stratification in patients with Ph-positive ALL [6, 7]. Distinct levels of the MRD load as assessed by RQ-PCR have been defined as guidelines for therapeutic decisions [19, 62]. Molecular diagnostics and immunophenotyping have become the basis for targeted therapy in ALL, as demonstrated by the use of tyrosine kinase inhibitors for *BCR-ABL1*-positive ALL, and rituximab for CD20-positive B-cell precursor ALL [16] or mature B-ALL/Burkitt lymphoma [19], which improved the prognosis of these previously highly adverse subtypes. Screening for *BCR-ABL1* mutations can be helpful to identify patients with Philadelphia-positive ALL who may have a benefit from second tyrosine kinase inhibitors or novel compounds targeting the T315I. Considering the recent introduction of high-throughput sequencing into hematological diagnostics [63], the potential of this novel technology should be explored for mutation screening, the definition of new therapeutic targets, and follow-up diagnostics in the acute lymphoblastic leukemias.
